# Obscure Gastrointestinal Bleeding and Capsule Endoscopy: A Win-Win Situation or Not?

**DOI:** 10.7759/cureus.27137

**Published:** 2022-07-22

**Authors:** Apurva Patel, Deepanjali Vedantam, Devyani S Poman, Lakshya Motwani, Nailah Asif

**Affiliations:** 1 Research, Gujarat Medical Education & Research Society Medical College and Hospital, Gotri, Vadodara, IND; 2 Internal Medicine, Kamineni Academy of Medical Sciences and Research Centre, Hyderabad, IND; 3 Research, Smolensk State Medical University, Smolensk, RUS; 4 Research and Development, Smt. Nathiba Hargovandas Lakhmichand Municipal Medical College, Ahmedabad, IND; 5 Research, Ras Al Khaimah College of Medical Sciences, Ras Al Khaimah, ARE

**Keywords:** gastrointestinal bleeding, cost-effectiveness analysis, upper gastrointestinal bleed, diagnostic yield and safety, patient preference, video capsule endoscopy, obscure gastrointestinal bleeding

## Abstract

Obscure gastrointestinal bleeding (OGIB) refers to bleeding of uncertain origin that persists or recurs after negative workup using any of the radiologic evaluation modalities. It can be divided into two types based on whether clinically evident bleeding is present, namely, obscure overt and obscure occult bleeding. As the visualization of the bowel mucosa is challenging, capsule endoscopy (CE) is the ideal go-to procedure as the process is wireless, ingestible, small, disposable, and, most importantly, non-invasive. This review article has compiled various studies to shed light on the guidelines for using CE, its structure and procedure, patient preferences, diagnostic yield, cost-effectiveness, and the future. The goal of this review is to show the influence of CE on OGIB on the aspects mentioned earlier.

## Introduction and background

Obscure gastrointestinal bleeding (OGIB) is commonly described as bleeding of an uncertain origin that persists or recurs (reported as recurrent or persistent iron-deficiency anemia (IDA), fecal occult blood test positivity, or visible bleeding) after a negative workup of esophagogastroduodenoscopy (EGD), colonoscopy, and radiologic evaluation of the small bowel, such as small bowel barium follow-through or enteroclysis [1]. Based on the presence or absence of clinically visible bleeding, it can be categorized as obscure overt and obscure occult bleeding, respectively [1]. Due to the recent advances in investigative procedures, such as video capsule endoscopy (VCE) and double-balloon enteroscopy (DBE), the delay in diagnosis has been overcome and has given us a chance to reconsider the conventional classification of gastrointestinal (GI) bleeding as upper or lower GI bleeding, depending on whether the bleeding is proximal or distal to the Treitz ligament [2]. It provides a better understanding of the involved portion by dividing GI bleeding (and OGIB) into three subgroups, namely, upper, middle, and lower GI bleeding, i.e., above the ampulla of Vater, from the ampulla of Vater to the terminal ileum, and after the terminal ileum, respectively [2]. The commonly used classification is based on the location with respect to the ligament of Treitz, i.e., proximally upper gastrointestinal bleeding (UGIB) and distally lower gastrointestinal bleeding (LGIB) [3,4]. LGIB is less prevalent than UGIB; therefore, the discussion of this review article focuses on the small bowel. The incidence of GI bleeding is higher in men than in women, which can be attributed to a higher prevalence of vascular diseases and diverticulosis among men [5]. Although UGIB cases are more prevalent than LGIB, the mortality rates of both UGIB and LGIB are similar [6]. The causes of GI bleeding as per the American Gastroenterological Association Institute technical review 2007 are listed in Table 1 [2].

**Table 1:** Etiology of obscure gastrointestinal bleeding. GI: gastrointestinal; NSAID: non-steroidal anti-inflammatory drugs

Upper GI and lower GI bleeding	Mid GI bleeding
Upper GI lesions	Age <40 years
Cameron erosions	Tumors
Fundic varices	Meckel’s diverticulum
Peptic ulcer	Dieulafoy’s lesion
Angiectasia	Crohn’s disease
Dieulafoy’s lesion	Celiac disease
Gastric antral vascular ectasia	Age >40 years
Lower GI lesions	Angiectasia
Angiectasia	NSAID enteropathy
Neoplasms	Celiac disease
	Uncommon
	Hemobilia
	Hemosuccus pancreaticus
	Aortoenteric fistula

Patients can present with hematemesis, suggesting the origin of bleeding to be proximal to the ligament of Treitz; or melena involving esophageal, gastric, or proximal small intestine bleeding; or hematochezia, suggesting the involvement of the rectum [7]. An assessment of the hemodynamic state, identification of the relevant risk factors, and proper triage of the level of care are the first steps of evaluation in a suspected OGIB case, followed by an endoscopic assessment that can be conducted after resuscitation measures [3]. Rebleeding is one of the critical risk factors and complications of OGIB, and Baba et al. proposed that chronic kidney disease, vascular abnormalities, and overt prior bleeding were all linked to a greater risk of rebleeding in univariate analysis, and these factors were observed to be an independent risk factor for rebleeding in a multivariate analysis [8]. Considering the other diagnostic modalities (push enteroscopy (PE), small bowel barium radiography, computed tomography angiography (CTA), computed tomography enterography (CTE), and magnetic resonance enterography), Singeap et al. reviewed the performance of small bowel capsule endoscopy (SBCE) as the first-line investigation for suspected small bowel pathologies, and OGIB is its most frequent indication [9].

Compared to the esophagus, stomach, and colon, the small bowel poses a challenge for endoscopic evaluation and therapy because of its length, angulated structure, and restricted equipment. Notably, SBCE, being wireless and minimally invasive, is the gold standard for the visualization of the small intestine [10]. In the case of OGIB, which is evaluated by various modalities, SBCE can be considered the best method for early diagnoses, good patient compliance, and potential savings. Obstruction, inability to perform tissue biopsy, and risk of perforation are not substantial limitations, which is an aspect to be considered for other pathologies, such as Crohn’s disease and neoplasms [9-11].

The primary goal of this review is to demonstrate the impact of VCE on OGIB diagnosis and management, including the following aspects: comparison to other modalities, patient acceptance, drawbacks, and cost-effectiveness.

## Review

Capsule endoscopy: overview

History: Origin to the Present Scenario

The capsule endoscope is a disposable, miniature, ingestible, wireless micro camera that allows direct viewing of the GI mucosa, making CE a simple and non-invasive test [12,13]. Recent major technological advancements, both in the capsule itself and the accompanying hardware and software, have dramatically increased the image quality and battery endurance in previous years. At present, four mega-companies manufacture capsule endoscopes: Given Diagnostic Imaging Ltd. (eventually purchased by Medtronic) (Israel), Olympus (Japan), IntroMedic (Korea), and Chongqing Jinshan Science and Technology Group (China) [14,15]. The difficulty in completely visualizing the intestinal mucosa with the existing endoscopic and radiographic procedures usually results in poor patient evaluation. However, it was not until Given Diagnostic Imaging Ltd., the first company to introduce a CE device named M2A (mouth to anus), was Food and Drug Administration approved in August 2001 [12-17]. Technological advancements have resulted in the creation of second- and third-generation capsule endoscopes, which overcome some of the limitations of the first-generation CE device by enhancing the field of vision, prolonging the effective battery life, and including a variety of other systems that provide higher resolution, more tissue coverage, and better analysis efficiency [16]. Subsequently, its second version, M2A plus, was launched, followed by the third version, the PillCam SB series, specifically for the small bowel with better resolution and an auto-adjustable frame acquisition speed [17]. Given Diagnostic Imaging Ltd. also introduced the PillCam ESO and PillCam Colon for esophageal and large bowel diseases, respectively [15]. Furthermore, Olympus (Japan) released the EndoCapsule, IntroMedic (Korea) released the MiroCam, and Chongqing Jinshan Science and Technology Group (China) released the OMOM capsule for the small bowel [15].

Structure and Mechanism of Capsule Endoscope

**Figure 1:**
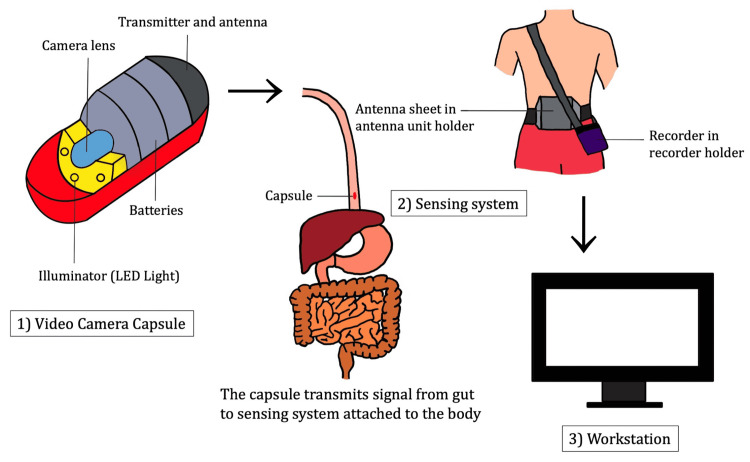
Video capsule endoscopy system. LED: light-emitting diodes Image credits: Apurva Patel.

The VCE system is composed of the following three parts: (1) a video camera capsule; (2) a sensing system, which consists of a data recorder, battery, and an array of antennas, encircling the body to receive the broadcast visual output; all the parts are mounted on a patient's belt, which holds the complete gadget; and (3) a workstation, which is a computer that processes and evaluates the downloaded pictures from the data recorder and converts them into a video data stream [15,17]. The three parts of the VCE system are shown in Figure 1.

A disposable plastic capsule, a complementary metal-oxide semiconductor or high-resolution charge-coupled device image-capturing system, a miniature lens, white-light-emitting diode lighting sources, and an internal power supply are the typical structural features of capsule endoscopes [18]. Either ultra-high-frequency band radio telemetry (PillCam, EndoCapsule, OMOM Capsule) or human body communications (MiroCam) is used to transmit data, whereas CapsoCam does not require a medium of communication as it is stored inside the capsule [14,18]. In the human-body communication approach, the capsule produces an electrical field that transmits data through human tissue, acting as a conductor [14,18]. As per the table showing the specifications of some CE systems, MiroCam, OMOM capsule 2, and CapsoCam SV-1 have demonstrated a longer duration than other capsule endoscopes, which can be beneficial in cases with delayed small bowel transit [14,17,18]. The photos were processed and delivered in single or multiple views at speeds ranging from five to 40 frames per second using proprietary software, which, along with video recordings, can be marked and saved [18]. All current software can recognize red pixels to aid in the diagnosis of bleeding lesions in the small intestine [18]. The localization of data, capsule transit progress inside the GI tract, and atlases to rapidly look upon and analyze report production are the other features provided [18]. All CE devices share approximately the same qualities and diagnostic capabilities as PillCam SB but differ in size, weight, photo capture rate, resolution, field of view, battery capacity, and image transmission technology, as depicted in Table 2 [14,15,17,18].

**Table 2:** Specifications of various capsule endoscopy systems.

	Manufacturer	Size (mm)	Weight (g)	Field of view (°)	Image/second	Battery life (h)	Resolution (pixels)
PillCam ESO 2	Medtronic	11 × 26	<4	169	18	8	256 × 256
PillCam SB 3	Medtronic	11 × 26	3.4	156	2–6	8	-
EndoCapsule	Olympus	11 × 26	3.8	145	2	8	512 × 512
MiroCam	IntroMedic	11 × 24	3.4	150	3	11	320 × 320
OMOM Capsule 2	Jinshan Science and Technology	11 × 25	4.5	165	2–6	≥10	-
CapsoCam SV-1	CapsoVision	11 × 31	3.8	360	12–20 (3–5/camera)	15	-

Guidelines

The number of suggestions issued by each of the three guidelines varied, ranging from two to 10 suggestions. There are two instances in which all the guidelines recommend CE as the initial diagnostic procedure in patients with OGIB, and in the case of unexplained IDA of unknown cause of bleeding, CE is the preferred method of evaluation. In addition, the Canadian Association of Gastroenterology (CAG) and the Korean Society of Gastroenterology (KSG) recommend CE as soon as possible after a recent episode of bleeding, unlike the European Society of Gastrointestinal Endoscopy (ESGE), which does not mention anything regarding this scenario. Among the three societies, KSG is the only one to provide guidelines for considering CE if there are other diagnostic modalities as part of the evaluation. Deep or flexible enteroscopy should be considered in patients with suspected stenosis, obstructive symptoms, or surgically altered anatomy according to the ESGE, ASGE, and British Society of Gastroenterology (BSG) recommendations (Table 3) [2,15,19].

**Table 3:** Various guidelines for obscure gastrointestinal bleeding. CE: capsule endoscopy; CTE: computed tomography enterography; CTEC: computed tomography enteroclysis; DBE: double-balloon enteroscopy; EGD: esophagogastroduodenoscopy; GI: gastrointestinal; IDA: iron-deficiency anemia; OGIB: obscure gastrointestinal bleeding; PE: push enteroscopy

Reference	Evidence assessment method/organization/guidelines	Statement	Strength of evidence	Strength of recommendation	Vote
Enns et al. (2017) [20]	The Grading of Recommendation Assessment, Development, and Evaluation (GRADE) and by voting of the consensus group of gastroenterologists practicing in Canada with expertise in the use of CE of the Canadian Association of Gastroenterology (CAG), which included six voting participants and a nonvoting moderator	In patients who have documented overt GI bleeding (excluding hematemesis) and negative findings on high-quality EGD and colonoscopy, we recommend CE as the next diagnostic step	Very low quality	Strong	Strongly agree, 100%
		In patients with an overt, obscure bleeding episode, we recommend that CE be performed as soon as possible	Very low quality	Strong	Strongly agree, 83%; agree, 17%
		In patients with prior negative CE who have repeated obscure bleeding, we recommend repeated studies (endoscopy, colonoscopy, and/or CE)	Very low quality	Strong	Strongly agree, 100%
		In patients with suspected obscure GI bleeding and unexplained mild chronic iron-deficiency anemia, we recommend that CE be used in selected cases	Low quality	Strong	Strongly agree, 50%; agree, 50%
Shim et al. (2013) [21]	Recommendations Assessment, Development and Evaluation Working Group and The Korean Society of Gastroenterology, The Korean Society of Gastrointestinal Endoscopy, and the Korean Association for the Study of Intestinal Diseases	CE is an effective initial diagnostic method for the evaluation of patients with OGIB.	Moderate	Strong	-
		CE is an effective initial diagnostic method for evaluating patients with IDA if no bleeding focus can be found outside the gastrointestinal tract	Moderate	Strong	-
		CE has a higher diagnostic yield than small bowel barium radiography in patients with OGIB	Moderate	Strong	-
		CE is more effective than enteroclysis in determining the cause in a patient with OGIB	Moderate	Strong	-
		CE could be more helpful than CTA in determining the cause of bleeding in a patient with OGIB	Low	Weak	-
		CTE/CTEC as a complementary examination to CE could be helpful in determining the cause of bleeding in patient with OGIB	Low	Weak	-
		CE has a higher diagnostic yield than PE in patients with OGIB	Low	Strong	-
		Performing CE as soon as possible in OGIB is effective in improving the diagnostic yield	Moderate	Strong	-
		CE and DBE provide similar diagnostic yields in patients with OGIB	Low	Strong	-
		It is recommended to perform CE prior to DBE for the diagnosis of patients with OGIB	Low	Strong	-
Ladas et al. (2009) [15]	The European Society of Gastrointestinal Endoscopy (ESGE) Guidelines Committee	VCE is the first-line examination in obscure gastrointestinal bleeding (OGIB) after a negative upper and lower gastrointestinal endoscopy	2b	B	-
		Patients with unexplained iron-deficiency anemia should undergo small-bowel VCE examination	2b	B	-

According to a study by Enns et al., the Grading of Recommendation Assessment, Development, and Evaluation, and by voting of the consensus group of the CAG, recommended that in documented overt GI bleeding patients, if findings are negative on EGD and colonoscopy, then CE should be performed. Similarly, CE must be performed immediately in patients with overt obscure GI bleeding [20]. Patients presenting with an episode of repeat obscure bleeding that was negative on CE before are required to undergo a repeat procedure, either endoscopy/colonoscopy or CE [20]. Until now, all guidelines mentioned had very low-quality evidence but a strong recommendation [20]. CE was recommended in selected cases in patients with suspected OGIB and unexplained long-standing mild IDA. This has low-quality evidence but a strong recommendation [20]. Two of the guidelines had strongly agreeing votes (100%), except the second and fourth guidelines mentioned in Table 3, which received the following votes: strongly agree, 83%; agree, 17%; strongly agree, 50%; and agree, 50% [20].

The KSG guidelines included various modalities other than CE, such as small-bowel barium radiography, enteroclysis, CTA, CTE/computed tomography enteroclysis (CTEC), PE, and DBE, stating that CE is superior to all modalities except DBE, with which it shares a similar diagnostic yield, and CTE/CTEC, to which it is a complementary tool [21]. It strongly recommends that CE is an effective initial diagnostic method for evaluating patients with OGIB with mild evidence; with the same strength of evidence and recommendation, it suggests that performing CE as soon as possible effectively improves diagnostic yield [21]. In addition, it strongly recommended with moderate evidence that in patients with IDA with no bleeding focus outside the GI tract, CE can be an effective initial diagnostic method (Table 3) [21].

Similar to the other two guidelines, the ESGE Guidelines Committee recommends CE as the first line of investigation after negative upper and lower GI endoscopy and in patients with IDA of unexplained origin (Table 3) [15].

According to the guidelines proposed by the ESGE, which were also reviewed and endorsed by the BSG, small-bowel CE should be performed as soon as possible after a bleeding episode, ideally within 14 days in patients with overt OGIB, to maximize the diagnostic yield (strong recommendation, moderate quality evidence) [19]. It also recommends small-bowel VCE as the first line of investigation in patients with OGIB (strong recommendation, moderate-quality evidence) [19]. According to a 2021 study by Chao et al. on the relationship between the timing of CE examination and the diagnostic correction rate in 60 patients with GI bleeding of unknown origin from a local hospital in Taiwan, CE examination should be performed within three days of the commencement of GI bleeding [22]. Another similar study by Kim et al. suggested that when VCE was completed within two days after the last overt OGIB, the diagnostic yield, therapeutic intervention rate, and hospital stay were all improved [23].

Bowel Preparation and Procedure

Bowel preparation is done by laxatives such as polyethylene glycol and/or prokinetics such as erythromycin, metoclopramide, and mosapride [14]. Although imaging encourages a 10-hour fast with no stool evacuation, some data show that polyethylene glycol, sodium picosulfate, sodium phosphate, or simethicone may improve imaging [24]. However, some have expressed concern that preparation fluids may dissipate any blood necessary to pinpoint a causative lesion in patients undergoing CE for OGIB [24].

Eight skin antennas were attached to the anterior abdominal wall of the patient and linked to the hard drive before ingestion of the capsule. After an eight-hour fasting period, the patient ingested the capsule along with water [12]. Drinking was permitted after two hours, and eating was permitted after four hours of capsule intake [12]. Upon releasing the capsule from its magnetic holder, the camera is turned on, and it starts capturing images per second and sends signals as per its medium of communication to the data recorder until the battery lasts [12]. For the following eight hours, the data recording device is linked to a workstation, which is a computer, where the data are retrieved, analyzed, and high-resolution endoscopic pictures and video data stream are displayed [25]. Within 24-48 hours, the capsule is excreted along with feces and is not reused [24,25].

Patient Acceptance/Preference

A study conducted in 2021 by Vuik et al. using PillCam COLON 2 on 451 participants concluded that colon capsule endoscopy (CCE) was safe and had good acceptance among patients [26]. CCE received a score of 7.8 on a scale of 1-10; additionally, 91.1% of the participants said they would consider having CCE sometime in the future [26]. Only 6.6% of those polled said that they would tell others to avoid CCE [26]. The most cumbersome element of the CCE process for most participants (89.2%) was bowel preparation, while for others it was the day of the procedure (8%) or stomach issues after the procedure (3%) [26].

Considering bowel preparation, capsule ingestion (relative to the placement of the tube/scope), stress of the complete procedure, duration, and compliance with pre-study instructions, CE was considerably superior to magnetic resonance enteroclysis and balloon-assisted enteroscopy (BAE) [27].

In a pilot study on the diagnosis of Barrett’s esophagus and esophageal varices, magnetically assisted capsule endoscopy (MACE) and gastroscopy were compared [28]. The MiroCam Navi capsule system was initially used, followed by gastroscopy [28]. On a 10-point scale, MACE was perceived as being more pleasant than traditional endoscopy (p < 0.0001); the mean score for MACE was 9.2 compared to 6.7 for gastroscopy [28]. Overall, MACE was found to be more comfortable and patient-friendly than gastroscopy but less accurate; therefore, it can be considered for screening [28].

Considering colon-related cases, CCE received 52% (95% confidence interval (CI) = 41-63) of the votes from patients, whereas conventional optical colonoscopy (COC) received 45% (95% CI = 33-57) of the votes, suggesting no significant difference between the two procedures [29]. CCE preference ranged from 13% to 82% in different studies, whereas the COC preference ranged from 18% to 69% [29]. The reasons for preferring CCE included lesser invasiveness, no requirement for sedatives or a driver, one investigation for a thorough check-up of all GI parts without the need for intravenous entry, lesser awkwardness and unpleasantness, maneuverability, availability to the investigation, and dread of COC-related unpleasantness or consequences [29].

Comparison With Other Modalities for Diagnostic Yield

All the studies mentioned in Table 4 mostly concluded that CE is superior to the other modalities, except for DBE and PE. CE did not show a statistically significant difference in the diagnostic yield between DBE and PE. Hence, it should be used in conjunction with them individually for routine workup. However, it is worth mentioning that the diagnostic yield of CE in patients with OGIB varies significantly not only in the studies stated here but also in many other publications (Table 4).

**Table 4:** Comparison of capsule endoscopy with other diagnostic modalities. CE: capsule endoscopy; MRE: magnetic resonance enteroclysis; BAE: balloon-assisted enteroscopy; DBE: double-balloon enteroscopy; CTEC: computed tomography enteroclysis; OGIB: obscure gastrointestinal bleeding; CD: Crohn’s disease; IOE: intraoperative enteroscopy; PE: push enteroscopy; CTA: computed tomography angiography; ANGIO: angiography; SMFT: small bowel follow-through

Reference	Design	Number of persons included	Diagnostic modality	Diagnostic criteria/Results	Conclusions
Wiarda et al. (2012) [30]	Prospective study	38	CE vs. MRE	BAE findings in visualized small-bowel segments and expert panel consensus for segments not visible during BAE served as the gold standard	CE outperformed MRE; however, MRE can be used in case of clinical signs of bowel constriction
Chen et al. (2007) [31]	Meta-analysis	277	CE vs. DBE	Fixed or random model method	The output of CE is more than DBE if oral and anal techniques are not combined, and in the combination, DBE is as effective as CE
Voderholzer et al. (2003) [34]	Prospective study	22	CE vs. CTEC	CE could diagnose four patients of OGIB, while CTEC diagnosed only one patient (p = 0.1)	CE detects more small-bowel lesions (OGIB, CD) than CTEC
Hartman et al. (2005) [36]	Prospective two-center study	47	CE vs. IOE	Patients with previous non-diagnostic assessment by upper endoscopy, colonoscopy, and PE, underwent CE preceded by IOE	CE is more effective at detecting bleeding sources in patients with OGIB. Henceforth, it should be part of the regular workup in OGIB patients
Saperas et al. (2007) [37]		28	CE vs. CTA CE vs. ANGIO	CE could detect the bleeding source in 72% of patients, CTA in 24%, and ANGIO in 56% of patients. CE could diagnose 12/19 negative cases on CTA and 6/11 cases negative on ANGIO	CE identified more lesions than CTA and ANGIO and showed therapeutic effects in half of the patients having positive outcomes
Costamagna et al. (2002) [39]	Prospective study	22	CE vs. SBFT	Compared to CE, barium SMFT had a substantially lower diagnostic potential	In assessing small bowel illnesses, CE was proven to be beneficial over SBFT
Sidhu et al. (2008) [40]		155	CE vs. PE	When patients who received CE followed by PE were compared to individuals devoid of CE, the diagnostic yield was 41% against 47% (P < 1).	PE provided the highest diagnostic yield in patients with overt bleeding. Henceforth, PE should be utilized in conjunction with CE for therapeutic purposes

In a prospective study by Wiarda et al., 38 patients aged 28-75 years were investigated using MRE, followed by CE if there was no stenosis on MRE and BAE [30]. BAE visualized small bowel segments, and expert panel consensus was used for non-visualized small bowel segments as diagnostic criteria [30]. Considering BAE as the standard, the diagnostic yields of MRE and CE were compared in patients with OGIB [30]. Out of 38 patients, four (11 %) had stenosis (n = 3; 8 %) or timing issues (11 %), and one patient was non-diagnostic for CE [30]. DAE could detect abnormalities in 20 (53 %) patients [30]. MRE showed the following values: sensitivity (21%), specificity (100%), positive likelihood ratio (infinity), and negative likelihood ratio (0.79) [30]. CE showed the following values: sensitivity (61%), specificity (85%), positive likelihood ratio (4.1), and negative likelihood ratio (0.46) [30]. It was concluded that CE did not deviate much from the reference standard (p = 0.34), while MRE varied considerably (p < 0.001) [30]. MRE was also used in cases where clinical signs of bowel constriction were suspected [30].

CE and DBE are considered acceptable diagnostic and therapeutic methods for small-bowel disorders and supportive treatments [31]. A meta-analysis of eight prospective studies by Chen et al., published in 2007 with 277 sample populations compared the diagnostic yields of DBE and CE considering the odds ratio (OR) of diagnostic yields of the two modalities to measure the outcomes [31]. The study showed no significant difference between CE and DBE (170/277 of CE and 156/277 of DBE, OR = 1.21 (95% CI = 0.64-2.29)) [31]. Subsequently, a sub-analysis was conducted, which found that DBE was performed via two different insertion approaches, namely, oral and anal, and when a combined approach was used, CE was not higher than DBE (26/48 of CE and 37/48 of DBE, OR = 0.33 (95% CI = 0.05-2.21), p > 0.05) [31]. In contrast, CE yielded drastically more than DBE when no combined approach was used (137/219 vs. 110/219, OR = 1.67 (95% CI = 1.14-2.44), p < 0.01) [31]. Similarly, a prospective single-center retrospective study suggested that both modalities had similar diagnostic yields, with DBE having therapeutic benefits [32,33].

In a prospective study conducted in 2003 by Voderholzer et al., over 22 patients with suspected bowel disease, including cases of OGIB (n = 8), Crohn’s disease (n = 8), unexplained diarrhea (n = 5), or suspected carcinoid tumor (n = 1), were investigated by two independent blinded investigators, and the results were compared and interpreted by a third investigator who concluded that CE was more efficient in detecting small bowel lesions than CTEC [34]. CE could detect lesions in 13 (59%) patients and CTEC in eight (36%) patients (p = 0.12) [34]. In seven patients (cases other than OGIB), no lesions were detected in the bowel using either method; however, OGIB patients were detected [34]. The diagnosis was established by CE in four patients with obscure bleeding, whereas CTEC was positive in only one patient (p = 0.1) [34]. In another study by Zhang et al., among 123 patients, CE detected OGIB in 71 (57.72%) patients (p > 0.05), and multiple-detector computed tomography (MDCT) detected OGIB in 37 (30.08%) patients (p < 0.01) [35]. The combined use of CE and MDCT had a higher detection value than MDCT alone, but not higher than the individual detection rate of CE [35]. Hence, there was a considerable difference in the detection rate between CE and MDCT; however, no significant difference was observed between CE + MDCT and CE alone [35].

In a prospective two-center study of 47 patients with OGIB from two German gastroenterology centers by Hartman et al., all previous non-diagnostic patients with ongoing overt bleeding, previous overt bleeding, or obscure-occult bleeding underwent CE followed by intraoperative enteroscopy (IOE) showed that CE detected 100% lesions in ongoing over bleeding patients and 67% lesions in the remaining two categories [36]. In 74.4% of all patients, CE revealed the cause of bleeding [36]. CE showed sensitivity, specificity, and positive and negative predictive values of 95%, 75%, 95%, and 86%, respectively [36]. Therefore, in patients with OGIB, CE can be a part of check-ups as it is superior to IOE in the detection of a bleeding source [36].

In a prospective study conducted by Saperas et al., 28 admitted patients with OGIB underwent CTA and angiography (ANGIO) first preceded by CE within seven days of admission, performed by blinded independent examiners, which resulted in significant bleeding source detection by CE, 72% (18/25, 95% CI = 50.6-87.9%) compared to CTA, 24% (6/25, 95% CI = 9.4-45.1%, p = 0.005 vs. CE) and ANGIO, 56% (14/25, 95% CI = 34.9-75.6%, p = NS) [37]. Furthermore, CE could detect the bleeding source in 12 of 19 (63%) CTA-negative patients and six of 11 (55%) ANGIO-negative cases [37]. Therefore, CE identifies more lesions than CTA and ANGIO [37]. In another randomized controlled study by Leung et al., 60 patients who presented with acute melena or hematochezia with previous non-diagnostic endoscopy were randomized for CE or ANGIO [38]. The diagnostic yield and long-term outcomes were compared [38]. The diagnostic yield of immediate CE was 53.3%, whereas that of ANGIO was 20% (p = 0.016) [38]. Long-term outcomes, such as rebleeding, transfusion, and death, did not differ significantly between the two techniques [38]. Thus, compared to ANGIO, instant CE provides a greater diagnostic yield than equivalent long-term results in individuals with overt OGIB [38].

For the assessment of small bowel illnesses, CE was proven to be beneficial over small bowel follow-through (SBFT) in a prospective study published in 2002 by Costamagna et al., including 22 patients (subsequently, two patients were excluded due to ileal stenosis) in whom the clinical outcomes of SBFT and CE were compared [39]. SBFT detected lesions in three patients and was normal in the other 17 patients, whereas CE showed contrasting results with detection in 17 patients and three control patients [39]. Four (20%) patients were diagnosed after SBFT, nine (45%) after CE, and eight (40%) showed suspected disease, whereas three (15%) showed negative outcomes [39]. The diagnostic yield of CE was 31%, whereas, for SBFT, it was 5% (p < 0.05) [39]. Hence, even with the need for further assessment, CE was observed to be superior to SBFT [39].

In contrast, another 2008 study by Sidhu et al., which included 155 patients, showed that PE provided the highest diagnostic yield in patients with overt bleeding compared with other indications of PE (overall diagnostic yield = 30%, p < 0.001) [40]. When performing CE followed by PE, the diagnostic yield was 41% compared to 47% in patients devoid of CE (p < 1) [40]. No single case was diagnosed only by PE and not by CE [40]. Compared to other indications, PE provided the highest diagnostic yield in patients with overt bleeding; therefore, PE should be used in conjunction with CE for therapeutic purposes [40]. However, this is not supported by a pilot study by Lewis et al., in which 21 patients were enrolled; PE showed a diagnostic yield of 30% and CE of 55%, which was not a significant statistical difference (p = 0.0625) [41]. CE detected a distal source of bleeding in five out of 14 patients where PE failed, and patients preferred CE over PE; thus, CE was better than PE as per the discussed aspects [41]. This observation was supported by another prospective controlled trial by Ell et al., who reported that CE could decrease the number of diagnostic procedures and be part of the initial workup, provided both upper and lower GI endoscopy is negative [42].

Cost-Effectiveness

According to a retrospective study conducted in 2007 by Marmo et al. on 369 patients with OGIB, CE is more cost-effective than other diagnostic methods. CE had a mean cost of €2090.76 for a positive diagnosis compared to a mean cost of €3828.83 for other procedures, representing a mean cost saving of €1738.07 (p < 0.001) for a single positive diagnosis [11]. A 2013 study by Meltzer et al. on patients who were brought to the emergency department with acute UGIB concluded that for low- and moderate-risk patients, VCE is the most cost-effective procedure (cost of $5,69-4.69 quality-adjusted life-years (QALYs) for low risk, cost of $9,190-14.56 QALYs for moderate risk) over nasogastric tube (cost of $8,159-14.69 QALYs for low risk, cost of $9,487-14.58 QALYs for moderate risk), risk stratification strategy (cost of $10,695-14.69 QALYs for low risk), and admit-all strategy (cost of $22,766-14.68 QALYs for low risk, cost of $22,584-14.54 QALYs for moderate risk) [43].

Contraindications and Drawbacks

CE allows complete imaging of the intestine. However, the presence of black or opaque intestinal contents and motility abnormalities might make a thorough inspection challenging because the intestinal mucosa is not visible [25]. According to the BSG and ESGE guidelines, in cases of obstruction and constriction, specialized small intestine cross-sectional imaging scans, such as MRE or CTE/CTEC, are initially employed (strong recommendation, low-quality evidence) [19]. Retention is a major drawback of CE in the case of Crohn’s disease or non-steroidal anti-inflammatory drug-induced enteropathy, but not much in the case of OGIB as it occurs with a variation from 0% in healthy people to 1.5% in patients with OGIB [1]. To deal with retention, Fairbrass et al. suggested a pre-test with a patency capsule before undergoing CE [44]. It cannot be used in patients with motility disorders for fear of retention, and even in patients who are allergic or have an MRE appointment within 14 days of ingestion of the capsule [26]. The main limitation of CE is that it is simply diagnostic, with no therapeutic benefits, such as conducting biopsies or delivering therapy, except for redirecting additional therapeutic steps [13].

Future and Implications

Since the first design of the capsule, manufacturers have introduced several improvements, such as increasing the sensitivity and specificity of the rapid identification of non-bleeding lesions, extending battery life, obtaining tissue samples, controlling widespread bowel disorders, and introducing capsule implantation and retrieval devices. In the pediatric population and patients with dysphagia or abnormal upper GI anatomy, swallowing becomes a hurdle. Therefore, the capsule can be placed endoscopically for which an endoscope delivery device named AdvanCE was used, which proved to be safe and easy to perform [24,45]. The use of portable belt recorders for a pediatric population also increased the feasibility of MRE being considered safe and reliable [24,45,46].

Considering the frames per second captured by different capsule endoscopes throughout 2-8 m of the gut, going through them becomes tiresome for the examiner considering the amount of attention required [47]. This can be resolved if machines learn the algorithm and detect abnormalities; thus, artificial intelligence (AI) was introduced [47]. From 2007 to 2020, AI has developed significantly in terms of performance by the mechanism for detecting pathologies (here color-coding to detect bleeding), type, sensitivity, specificity, accuracy, low computational cost, reporting time, and applicability in daily practice [48]. However, one of the future challenges AI faces is to develop a methodology for quantifying the uncertainty of AI, which considers both the dataset and the reliability of the inner algorithmic intricacies [48,49].

Until AI is developed to establish itself with full potential in daily practice, increments in the workforce, such as an endoscopy nurse, can be considered. Handa et al. considered the reading time and detection rate of significant lesions in CCE images as parameters for judging inexperienced nurses, expert endoscopy nurses, and inexperienced physicians [50]. The median reading time was shorter for expert endoscopy nurses (19 min) than for inexperienced nurses (45 min), with more thumbnails by expert endoscopy nurses than the rest [50]. Hence, expert endoscopy nurses can save physicians’ time with an enhanced diagnostic output [50]. Two similar studies concluded that expert endoscopy nurses could accurately detect and interpret lesions, limiting the physician’s role to simply confirming the thumbnails or looking into a pre-evaluated video [51,52].

Limitations

This review article focused primarily on CE for the small intestine rather than the esophagus and colon. CE effects should be compared further with DAE as both modalities share an almost exact diagnostic yield. CE also requires additional research in the case of guidelines with strong recommendations for several statements but poor quality of evidence. Moreover, it is a rapidly advancing technology; therefore, further research on this technology is needed.

## Conclusions

As evidenced by the findings discussed in this article, CE is the ideal investigative procedure to diagnose OGIB, despite the presence of other modalities. In terms of patient preference, diagnostic yield, and cost-effectiveness, which are fundamental factors for implementation, CE overshines other investigatory modalities in the case of OGIB. However, only DAE could provide tough competition to CE. In summary, the clinical implication of this review article is to establish a strong link between CE and OGIB, with the former being an inevitable investigation in the case of the latter, considering various day-to-day essential factors for its implementation. Hence, it can be said that it is a win-win situation. This article highlights that CE is the preferred method of investigation among patients. We believe that this article will enable doctors to consider CE as a leading and reliable investigative method for diagnosing OGIB. We highlighted the challenges faced and provided solutions to them. For instance, in cases where a stricture is suspected, a patency capsule can be used, and in patients with an inability to swallow, the capsule can be placed endoscopically. Lastly, we feel that the role of CE in the case of OGIB requires more in-depth research to develop an organized procedure to diagnose and manage OGIB.
